# An unusual distal tibial metastasis from urothelial carcinoma: A case report

**DOI:** 10.14440/bladder.2024.0077

**Published:** 2025-03-21

**Authors:** Tran Ngoc An Huynh, Darren Trinh, Janan Chandrananth, James Sewell

**Affiliations:** 1 Department of Urology, Monash Health, Melbourne, Victoria 3806, Australia; 2 Department of Orthopedics, Peninsula Health, Melbourne, Victoria 3199, Australia; 3 Department of Medicine, Faculty of Medicine, Nursing and Health Science, Monash University, Melbourne, Victoria 3800, Australia

**Keywords:** Bladder cancer, Urothelial carcinoma, Bone metastasis, Distal tibia

## Abstract

**Background:**

Bladder cancer (BCa) frequently metastasizes to bones, typically affecting the spine and pelvis.

**Case presentation:**

Presented here is a case of rare extensive distal tibia metastasis in an 84-year-old female with invasive high-grade urothelial carcinoma, who suffered from rapidly progressive ankle pain and reduced mobility. Imaging and biopsy confirmed metastatic urothelial carcinoma in the tibia, which was managed with surgical fixation and palliative radiotherapy. Although bone metastases in BCa commonly affect other sites, clinicians should consider atypical locations like the tibia in patients with unexplained bone pain.

**Conclusion:**

Early diagnosis and intervention are essential for improving patient outcomes and quality of life.

## 1. Background

Bladder cancer (BCa) is a prevalent urological malignancy that results in roughly 213,000 deaths worldwide each year.^1^ The most common sites of metastasis are the lymph nodes and bones, with the pelvis and spine being common locations for bone metastasis (BM).^2^,^3^ This was the first case report, to our knowledge, to describe a BM in the distal tibia from BCa.

## 2. Case presentation

An 84-year-old female was referred by an urologist to a secondary urologist for consideration of a robotic partial cystectomy following the diagnosis of invasive high-grade urothelial carcinoma within a bladder diverticulum. The diagnosis was made based on a transurethral resection of bladder tumor (TURBT) performed after she presented with hematuria. Her hemoglobin levels and renal function were within normal limits. A computed tomography (CT) scan of the chest, abdomen, and pelvis revealed thickening of the left bladder wall, with no hydronephrosis and no evidence of metastatic disease.

A repeat rigid cystoscopy performed 4 weeks after the initial TURBT showed multifocal recurrence along the left posterior and lateral bladder wall. The patient then underwent a TURBT, which revealed invasive (pT1) high-grade papillary urothelial carcinoma with 30% glandular differentiation.

**Figure 1:**
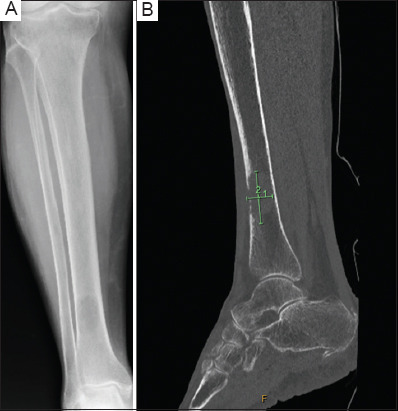
A lesion in the distal tibia of the right leg. (A) X-ray scan of the right lower leg (anterior-posterior view) showing a lesion in the distal tibia. (B) Non-contrast computed tomography scan of the right lower leg (sagittal view) confirms a lesion of 41 × 20 mm with a permeative breach of the anterior cortex

**Figure 2:**
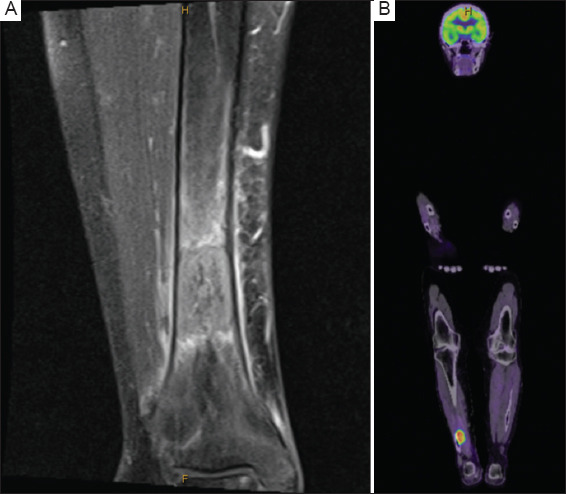
MRI and FDG PET scan of the patient. (A) An MRI scan of the right lower leg (coronal view) of the patient. The T1-weighted, contrast-enhanced, fat-saturated sequence demonstrates lesion and periosteal soft tissue enhancement. (B) An FDG PET scan (fused coronal view) of the patient Abbreviations: MRI: Magnetic resonance imaging; PET: Positron emission tomography; FDG: Fludeoxyglucose F18

Perioperatively, she reported worsening pain in her right ankle and left buttock over 4 weeks, leading to reduced mobility. X-ray of her right leg demonstrated a lesion in her distal tibia (Figures 1A). Magnetic resonance imaging (MRI) of her right ankle and further a CT scan revealed a 41-mm intramedullary lobulated mass in the distal tibia with contrast enhancement (Figures 1B and 2A). In addition, a fludeoxyglucose F18 positron emission tomography (PET) identified BM in the left distal sacrum and the distal right tibia (Figure 2B). A CT-guided biopsy of the tibial lesion confirmed metastatic carcinoma of urothelial origin.

The patient, with a Mirels score of 11, underwent prophylactic embolization and surgical fixation of the distal tibia. In addition, she received palliative radiotherapy to both the sacrum and tibia, which initially provided pain relief and improved mobility for 3 weeks. Unfortunately, a follow-up X-ray taken after 6 weeks showed that the lesion had grown to 150 mm. Due to the patient’s frailty, systemic chemotherapy was deemed unsuitable, and she transitioned to palliative care.

## 3. Discussion

BM in BCa occurs through several processes, including epithelial-to-mesenchymal transition, angiogenesis, intravasation, extravasation, and interactions with the bone microenvironment.^4^ The common sites of BCa-related BM are the spine, pelvis, ribs, skull, femur, and proximal end of the humerus.^4^

According to the European Association of Urology 2024 guidelines, integrating multimodal imaging enhances diagnostic accuracy for detecting BCa metastases.^5^ Plain radiographs are useful for identifying compression fractures and osteolytic metastases but are less reliable for spotting osteoblastic lesions. CT and MRI provide superior accuracy for identifying metastases and differentiating benign from malignant lesions. PET is valuable for assessing early tumor formation and metabolism, offering greater differentiation between benign and malignant lesions; however, it lacks precise anatomical resolution.^4^

The management of BM focuses on inhibiting disease progression and alleviating symptoms. Radiotherapy serves as a palliative, non-invasive treatment and typically improves pain within 2 – 6 weeks following therapy.^4^ Surgical intervention prevents or stabilizes fractures, preserves mobility, and relieves bone pain.^4^ Mirels proposed a scoring system that assesses the risk of fracture based on four factors: the location of the lesion, the nature of the lesion (lytic vs. blastic), its size, and the presence of pain.^6^ According to Mirels’ criteria, prophylactic fixation is recommended when a lesion scores 9 or higher, indicating a high risk of fracture.^6^

Brennan *et al*.^7^ described a 61-year-old male with a proximal tibial metastasis secondary to BCa, which was successfully managed with a total knee replacement and chemotherapy. Similarly, Kastanis *et al*.^8^ reported a 58-year-old male with metastasis to the tibial shaft, treated with intramedullary nailing for structural stabilization. To our knowledge, the present case is the first reported instance of a distal tibia BM from BCa.

## 4. Conclusion

Although bone is a common site for metastasis in BCa, clinicians should maintain a high index of suspicion for atypical sites of BM in patients presenting with unexplained pain and impaired daily functioning. As these sites are outside the standard imaging template for staging scans, recognizing the possibility of tibial BM in BCa is crucial for timely diagnosis and intervention.

## Data Availability

All data generated or analyzed during this study are included in this published article.
